# Prognostic Value and Therapeutic Potential of Novel Markers and Pathways in Oral Squamous Cell Carcinoma

**DOI:** 10.3390/biomedicines14030536

**Published:** 2026-02-27

**Authors:** K. Devaraja, Sadhna Aggarwal

**Affiliations:** 1 Department of Head and Neck Surgery, Kasturba Medical College, Manipal Academy of Higher Education, Manipal 576104, India; 2 Department of Radiation Oncology, The University of Texas MD Anderson Cancer Center, Houston, TX 77030, USA

The articles published in this Special Issue of *Biomedicines*, titled ‘Oral Squamous Cell Carcinoma (OSCC): Molecular Signaling Pathways and Novel Biomarkers,’ thoroughly explore some novel molecular biomarkers and signaling pathways that exhibit either proven or potential diagnostic/prognostic value in OSCC, with or without an additional therapeutic implication. This Editorial reiterates some of the highlights published in this unique collection, which has positioned itself at the intersection of molecular research and its translation to clinical care.

The article by Tsai et al., from Chang Gung Memorial Hospital, Taiwan, evaluated the prognostic significance of total cholesterol (TC) in a retrospective cohort of surgically treated OSCC [1]. Of the 331 patients undergoing ablative surgery over 10 years, 310 previously untreated cases fulfilled eligibility criteria, and in these patients, the majority were stage IV and had thus received multimodal treatment. Through receiver operating characteristic curve analysis, the authors found 157 mg/dL as the optimal cut-off value of TC to predict the overall survival (sensitivity = 80.4%; specificity = 43.7%). Along with the other ‘known’ predictors of overall survival, such as advanced stage, presence of perineural invasion, lymphovascular invasion, poor differentiation, and close resection margin, a low TC (<157 mg/dL) also demonstrated its independent prognostic capabilities for predicting overall survival, both on univariable Cox analysis and multivariable Cox regression analyses. Similar associations were also noted between low TC and disease-free survival. The authors of this study have also constructed a nomogram to predict survival based on these prognostic factors and found their nomogram to have a superior discriminative ability over the TNM classification of the VIII edition of the *American Joint Committee on Cancer Staging Manual*. Nevertheless, the results of this study not only warrant further large and prospective studies to pursue the exact role of pre-treatment TC in predicting the prognosis in OSCC but also provide impetus to design interventional studies to evaluate the efficacy of lipid-modifying drugs in imparting better prognosis [2].

A review by European authors exploring the etiopathological role of dental trauma in OSCC is another fascinating article published in this Special Issue [3]. By reviewing 33 original studies, this scoping review sheds light on some interesting findings. As per this review, there is an etiopathological link between chronic mucosal trauma and oral cancer, particularly of the lateral tongue. It was further observed that younger patients and the presence of sharp teeth were associated with oral tongue lesions, and older patients and denture friction were found to be associated with lesions in the gingiva, floor of the mouth, and buccal mucosa. Further, this review also discusses the nuanced association between oral hygiene and OSCC, including the potential role of the altered oral microbiome in oral carcinogenesis. As per this review, which aligns with other recent reviews on the topic, the OSCC was associated with a typical microbial profile that demonstrates the increased abundance of genera *Fusobacterium*, *Peptostreptococcus*, *Neisseria*, and *Parvimonas*, with or without a significant reduction in commensals, such as *Streptococcus*, *Rothia*, *Actinomyces*, and *Megasphaera* [3,4]. However, to date, there is no direct evidence to establish the causality between oral dysbiosis and OSCC [4]. The authors of this review acknowledge the need for further research on these topics to provide more robust evidence on the link between chronic mucosal irritation caused by sharp teeth or dentures, the oral microbiome, and oral carcinogenesis.

This Special Issue contains four other original studies and a review that explore molecular pathways and novel markers and their clinico-pathological and therapeutic implications.

Dr Subhanwita Sarkar and colleagues from the Universities of Calgary and Toronto, Canada, present their findings of in vitro and in vivo investigations that were aimed at evaluating the role of an inhibitor of polo-like-kinases-1 (PLK-1) in potentiating the therapeutic effect of radiotherapy in OSCC [5]. For the in vitro experiment, the authors used commercially available OSCC cell lines, gene expression data, and clinical outcome data, downloaded from The Cancer Genome Atlas (TCGA) database and Sage Synapse, respectively. For in vivo assessment, they used a mouse model (n = 22), which involved the induction of tumors in all mice and delivering separate treatment to four cohorts of mice in the form of ionizing radiation (n = 7); volasertib, a second-generation PLK-1 inhibitor (n = 5); neither of the two (n = 3); and both ionizing radiation and volasertib (n = 7). In the in vitro studies, most of the OSCC cell lines were highly sensitive to volasertib, with near-complete resistance in some; however, this sensitivity pattern paralleled the survival outcomes, suggesting that personalized therapy based on molecular markers could improve prognosis in OSCC. The combination therapy (radiotherapy followed by volasertib) exhibited more anti-tumor effects than either volasertib or radiation alone, both in the in vivo and in vitro experiments. The higher therapeutic efficacy of the combination was also supported by anti-proliferative molecular changes observed in the corresponding OSCC cell lines. Though further studies are warranted to establish its clinical translation, this is one of the first studies to have evaluated the therapeutic potential of PLK-1 inhibitors in augmenting the efficacy of radiotherapy in OSCC.

Another molecular study, authored by Dr. Kendra Smith et al., examined the expression of a novel tumor suppressor gene, Krüppel-type zinc finger protein (ZNF671), in an independent patient cohort of the TCGA and found it to be epigenetically silenced and hypermethylated in a significant number of head and neck squamous cell carcinoma (HNSCC) tumor tissue samples, including those arising in the oral cavity, as compared to the matched adjacent normal tissue from the same patients [6]. Further, in their subgroup analysis, patients whose primary tumors expressed low levels of ZNF671 showed significantly decreased survival compared to the rest of the cohort, and this paralleled their in vitro analysis, wherein the overexpression of ZNF671 in HNSCC cell lines was associated with a significant reduction in tumor cell mobility and invasion compared to the empty-vector control cells. Encouraged by these preliminary findings on the role of ZNF671 in head and neck carcinogenesis, the authors of this study are undertaking further studies to understand the downstream genes and molecular mechanisms that regulate ZNF671 expression in HNSCC [6].

A group of authors from the USA published results of a pre-clinical test in which they investigated the impact of ‘stimulator of interferon genes (STING) agonism’ on cancer-associated pain in an OSCC model [7]. The results of this paper could lay the foundation for new therapeutic strategies in the form of STINGel (an extended-release formulation that prolongs the availability of STING agonists), not only for its antinociception ability that is crucial for cancer pain management but also for its anti-tumorigenic impact. Similarly, a team of scientists from Showa University, Tokyo, Japan, under the primary authorship of Dr. Masataka Watanabe, investigated the role of tumor protein (TP)D53, a member of the TPD52 family, in the malignant transformation of low-grade malignancy OSCC cells [8]. This was the first study to have studied the role of TPD53 in oral carcinogenesis. Here, the results of both in vivo and in vitro experiments suggested the possibility of an active role of TPD53 in the progression of low-grade OSCC, prompting further studies to investigate the underlying signaling pathways, as well as the therapeutic potential of targeting this novel protein.

Lastly, a narrative review by researchers at the Stem Cell and Cancer Research Lab of Amity University Uttar Pradesh, India, provides a detailed overview of the therapeutic utility of cancer stem cell (CSC)-derived exosomes in OSCC [9]. While CSCs comprise a small proportion of tumor cells able to self-renew and differentiate, the exosomes are small extracellular vesicles that facilitate intercellular communication by transferring proteins, lipids, and nucleic acids (Yang). CSC-derived exosomes have already been identified to play a crucial role in almost all cancer hallmarks, including immune evasion mechanisms, but its association with OSCC is not yet fully understood [10]. This review by Dr Prabhat Kumar and colleagues, after carefully appraising the relevant experiments and in vitro studies on CSC-derived exosomes, underscores the multifaceted roles of CSC-derived exosomes in OSCC, highlighting their potential as therapeutic targets and biomarkers for therapeutic response and disease progression [9].

Overall, this Special Issue of biomedicines presents original studies and reviews that provide insight into several novel biomarkers and signaling pathways involved in OSCC, emphasizing their prognostic capabilities and potential therapeutic propositions. By providing a brief synopsis of clinical correlations with biomarkers like ZNF671, STINGgel, TPD53, CSC-derived exosomes, and TC, the articles in this collection are expected to attract further larger studies to externally validate these results and translate them into clinical practice.
